# Drivers of retention of the HIV workforce transitioned from PEPFAR support to the Uganda government payroll

**DOI:** 10.1186/s12960-023-00824-6

**Published:** 2023-05-09

**Authors:** Henry Zakumumpa, Joseph Rujumba, Marjorie Kyomuhendo, llyse Stempler, Woldekidan Amde

**Affiliations:** 1 School of Public Health, Makerere University, Kampala, Uganda; 2 College of Health Sciences, Makerere University, Kampala, Uganda; 3 College of Humanities and Social Sciences, Makerere University, Kampala, Uganda; 4 Open Development LLC, Washington, DC United States of America; 5 School of Public Health, University of the Western Cape, Cape Town, South Africa

**Keywords:** HIV, PEPFAR, Human resources for health, Implementation research, Health systems, Resource limited settings, Health workforce, Uganda

## Abstract

**Background:**

Health worker (HW) retention in the public health sector in Uganda is an enduring health system constraint. Although previous studies have examined the retention of in-service HWs, there is little research focusing on donor-recruited HWs. The objective of this study was to explore drivers of retention of the HIV workforce transitioned from PEPFAR support to the Uganda government payroll between 2015 and 2017.

**Methods:**

We conducted ten focus group discussions with HWs (*n* = 87) transitioned from PEPFAR support to the public sector payroll in 10 purposively selected districts across Uganda. In-depth interviews were conducted with national-level stakeholders (*n* = 17), district health and personnel officers (*n* = 15) and facility in-charges (*n* = 22). Data were analyzed by a hybrid approach of inductive and deductive thematic development based on the analytical framework by Schaefer and Moos regarding individual-level and organizational-context drivers.

**Results:**

At the individual level, job security in the public sector was the most compelling driver of health worker retention. Community embeddedness of HWs in the study districts, opportunities for professional development and career growth and the ability to secure salary loans due to ‘permanent and pensionable’ terms of employment and the opportunity to work in ‘home districts’, where they could serve their ‘kinsmen’ were identified as enablers. HWs with prior private sector backgrounds perceived public facilities as offering more desirable challenging professional work. Organizational context enablers identified include perceptions that public facilities had relaxed supervision regimes and more flexible work environments. Work environment barriers to long-term retention include frequent stock-out of essential commodities, heavy workloads, low pay and scarcity of rental accommodation, particularly in rural Northern Uganda. Compared to mid-cadres (such as nurses and midwives), higher calibre cadres, such as physicians, pharmacists and laboratory technologists, expressed a higher affinity for seeking alternative employment in the private sector in the immediate future.

**Conclusions:**

Overall, job security was the most compelling driver of retention in public service for the health workforce transitioned from PEPFAR support to the Uganda government payroll. Monetary and non-monetary policy strategies are needed to enhance the retention of upper cadre HWs, particularly physicians, pharmacists and laboratory technologists in rural districts of Uganda.

## Background

Sub-Saharan Africa (SSA) has only 3% of the global health workforce despite having 25% of the world’s disease burden [1]. As is the case in many countries in SSA, the health system in Uganda is confronted with a myriad of health workforce constraints, prominent among which is the attrition of health workers and the inability to retain qualified personnel, which contributes to persistent staffing gaps at almost all levels of the health system [2]. Over the past two decades, health workforce bottlenecks have emerged as a fundamental barrier to achieving universal access to anti-retroviral therapy (ART) [3].

Uganda is heavily dependent on global health initiatives (GHIs), notably President’s Emergency Plan for AIDS Relief (PEPFAR) and the Global Fund for AIDS, Tuberculosis, and Malaria (Global Fund) in its national HIV response [4]. Since 2004, PEPFAR has supported Uganda’s national HIV response by rapidly expanding ART coverage and rolling out HIV prevention initiatives, such as safe male circumcision (SMC) and pre-exposure prophylaxis (PrEP) [2–7].

Although PEPFAR initially focused on supporting Uganda’s emergency response to the HIV epidemic, especially through the roll-out of ART country-wide, it became evident that health system constraints would remain a fundamental impediment to attaining UNAIDS’ 95–95–95 targets in Uganda [5, 6].

Within the cluster of health-system bottlenecks, health workforce shortages were a fundamental impediment to attaining HIV services scale-up goals. For instance, Uganda is listed by the World Health Organization (WHO) among the 57 countries with a crisis in human resources for health (HRH), with one health worker per 700 people, falling below the WHO minimum threshold [2].

To achieve HIV epidemic control, PEPFAR supported the recruitment of critical health workers (HWs) to boost the Government of Uganda (GoU)’s efforts to strengthen the HIV workforce [6].

### PEPFAR HIV workforce expansion intervention

Between 2012 and 2017, PEPFAR recruited 3 154 additional HWs through a cooperative agreement with the GoU and implementing partners (IPs) and seconded them to the Ministry of Health (MoH) headquarters, central institutions, such as Central Public Health Laboratories (CPHL), AIDS Control Program (ACP), National Referral Hospital, Regional Referral Hospitals (RRHs), District Health Offices, District hospitals and lower-level health facilities across the country [6].

At the level of district hospitals, which was the focus of our study, the cadres of HWs seconded through a competitive selection process included physicians, clinical officers, midwives, nurses, pharmacists and laboratory technologists [6]. Under the cooperative agreement, PEPFAR paid the salaries of the selected personnel for a minimum of 2 years, while they served in the district hospitals, with the understanding that they would subsequently be absorbed on the GoU payroll as soon as fiscal space allowed. The PEPFAR-recruited HIV workforce was paid salary scales comparable to those of similar rank in GoU service. In addition, they were paid a monetary housing allowance on top of their monthly salary [6]. The recruitment of HWs was premised on the GoU’s commitment to prioritize the absorption of this expanded workforce onto the GoU payroll when it became feasible to ensure the sustainability of the national HIV response [6]. After the initial 2-year contract phase, PEPFAR supported the recruitment processes of the seconded HWs, such as payment of monetary allowances for district personnel officials to convene formal recruitment meetings at sub-national level in Uganda [6].

A recent study found that between June 2015 and December 2017, of the PEPFAR-recruited HIV workforce, 694 were absorbed into mainstream GoU employment (Table 1) [6].

**Table 1:** Cadres of health workers transitioned from PEPFAR to Government of Uganda

Health worker cadre	No. of transitioned HWs	% by HW cadre *N* = 694
Enrolled Nurse	275	39.6
Enrolled Midwife	204	29.4
Medical Laboratory Technician	54	7.8
Medical Clinical Officer	50	7.2
Biostatistician	35	5.0
Medical Officer	30	4.3
Nursing Officer Nurse	14	2.0
Medical Laboratory Technologist	13	1.9
Enrolled comprehensive Nurse	8	1.2
Nursing Officer Midwife	6	0.9
Dispenser	3	0.4
Pharmacist	1	0.1
Medical Records Assistant	1	0.1
Laboratory Assistant	0	0.0
Anesthetic Officer	0	0.0
Total	694	100

PEPFAR plans to transition all HWs it recruited under the intervention to the GoU payroll to support accelerated HIV service delivery across Uganda in line with UNAIDS’s 95–95–95 targets [7]. However, there is little empirical understanding of the drivers of retention of HWs once they are absorbed into the Ugandan public service, given their diverse work backgrounds. Health worker retention is the decision to stay in practice [8].

Although there is a considerable body of evidence assessing determinants of retention of in-service HWs, especially those in the public sector [9–12], there is a dearth of evidence on the retention of a workforce which was initially donor-recruited and salaried before subsequently transitioning to the public sector [13]. The PEPFAR-recruited workforce had diverse work backgrounds, some of the HWs were recruited from the private sector with differing salary scales, work environments and organizational cultures. A previous study found that 694 HWs recruited with PEPFAR support had been absorbed into the GoU payroll [6].

Retaining skilled and experienced HWs is critical in sustaining gains in HIV epidemic control [14]. Previous studies on HW retention, in general, suggest that financial rewards, recognition, and opportunities for further education, promotion, and career development influence HW retention [9–12]. A study conducted in Tanzania on turnover intentions among nurses providing prevention-of-mother-to-child-transmission of HIV (PMTCT) services revealed that 35% of the nurses interviewed expressed an intention to leave service owing to job stability dissatisfaction and a lack of recognition and feedback from superiors [15]. These studies suggest that individual and contextual factors could be at play in facilitating or hindering the retention of health workers [9–12]. However, the aspects likely to influence the retention of transitioned HWs from PEPFAR to GOU service must be better understood. Yet, these factors have implications for effective human resources for health planning in the context of donor transitions [16]. Furthermore, there could be implementation research ‘lessons learned’ for the benefit of various stakeholders, including national governments and external donors involved in future HW transition processes that are worthwhile for Uganda and other countries that depend on external assistance in expanding their health workforce.

The objective of this study was to explore drivers of retention in public service of the health workforce transitioned from PEPFAR support to the Uganda government payroll.

## Methods

### Research design

We report qualitative findings from a larger mixed-methods study [6, 17]. The parent study entailed a secondary analysis of data sets in the Uganda Human Resources Information System (HRIS) to assess the extent of absorption of HWs recruited by PEPFAR between 2015 and 2017 and subsequently enrolled on the Ugandan public sector payroll [6]. This paper describes the drivers of retention in the Ugandan public service for this absorbed workforce which was previously PEPFAR-salaried with a section of them having private sector backgrounds.

### Analytical framework

We adopted an analytical framework proposed by Schaefer and Moos [18] regarding the micro or individual-level and meso or organizational-level [19] determinants of workforce retention. According to this analytical framework, the organizational system entails: organizational cultures, physical environments, structures, policies and work climate, which influence workforce retention. The personal factors implicated by this framework include; the characteristics of individuals, such as their position at work and level of experience, socio-demographic characteristics, expectations and preferences about the workplace environment. In addition, this framework posits a dynamic interaction between individual-level attributes and organizational context factors influencing coping responses and employee retention outcomes*.* The analytical framework served as a broad deductive framework during data analysis and presentation of findings.

### Study sites and sample selection

The detailed sample selection criteria is described elsewhere [6]. However, for completeness, we purposively sampled 10 (out of 87) districts in Uganda which benefited from a PEPFAR intervention to expand the HIV workforce at district hospitals between 2013 and 2017 [6]. We purposively selected eight districts with the highest rates of absorption of HWs (Table 2). In addition, we purposefully selected two districts with the lowest absorption rates based on Uganda’s major geographic sub-regions [6]. We visited 10 district hospitals across Uganda, which absorbed HWs who were previously PEPFAR-supported. The district hospitals were spread across all of Uganda’s eight major geographic sub-regions as defined by the Uganda Bureau of Statistics [3]. Districts with the highest concentration of absorbed health workers and those with a relatively high HIV burden (which was PEPFAR’s focus) were purposively selected (Table 2).

**Table 2:** Characteristics of case-study districts

Geographic Sub-region of Uganda	District	HIV prevalence	Inclusion criteria
South Western	Sheema	8.0	High number of transitioned HWs and high HIV prevalence
	Bushenyi	6.8	Had lowest number of HWs absorbed on payroll (only one)
East Central	Iganga	4.4	High number of transitioned HWs, mixed rural–urban
Mid-East	Tororo	4.8	High number of transitioned HWs and cross border dynamics, mainly urban
Mid-West	Kasese	5.5	High number of transitioned HWs and cross border dynamics, mainly urban
Central 2	Mubende	7.4	High number of transitioned HWs and high HIV prevalence
Central 1	Nakaseke	6.9	Had lowest number of HWs absorbed on payroll (only one)
Mid North	Nwoya and Apac	7.0	High number of transitioned HWs and high HIV prevalence, largely rural
North East	Napac	3.4	High number of transitioned HWs and hard to reach, rural
Kampala	Kampala	6.6	Capital city and houses many agencies involved in transition planning

### Data collection

#### Focus groups discussions

We aimed to explore HW experiences, preferences and perspectives regarding retention in the Ugandan public health sector from the lens of Schaefer and Moos’ [18] analytical framework. We sought to understand HW experiences of retention in public hospitals *collectively as a group* to explore themes that cut across multiple HWs and deemed focus group discussions (FGDs) worthy of achieving this objective. To this end, we conducted one mixed-gender FGD in each of the ten case-study districts (Table 2). Each of the focus groups had between 8 and 13 participants. We conducted one focus group in each of the study districts to probe for differences in contexts (e.g., rural/urban setting, social amenities and living conditions etc.). We conducted ten FGDs in selected districts (Table 2). The inclusion criteria included; (a) HWs who were recruited under the PEPFAR health workforce expansion intervention between 2013 and 2017, (b) HWs who had been formally absorbed onto the GoU payroll and (c) HWs who had been in GoU for at least 3 years. Overall, 87 HWs participated in our ten focus groups. An approved topic guide was structured around the themes suggested by the adopted analytical framework of the study [18], which posits that individual-level and organizational context influences employee retention outcomes.

The FGDs were conducted by HZ and JR, who have extensive qualitative health services research backgrounds. Two Research Assistants supported the two investigators, who took notes during the proceedings and operated the recorder. The in-person focus groups were conducted on-site at district hospitals in the ten study districts. FGDs were conducted between June and September 2018 (round 1) and January to March 2020 (round 2) [6].

#### In-depth interviews

Beyond the perspectives of HWs, we sought to enact a complete picture of retention drivers from an analytical dimension of *actor*s, *context, content* and *process* proposed by Walt and Gilson [20]. To this end, we conducted 22 face-to-face interviews with facility in-charges in the ten district hospitals. At the sub-national level, we interviewed 15 District Health Officers (DHOs) and district personnel officers in the ten districts that absorbed the PEPFAR-supported HWs and had valuable ‘insider’ insights into the processes involved in absorbing PEPFAR-recruited workforce in the Ugandan public service At the national level, we conducted face-to-face interviews with 14 representatives of three sector ministries which participated in HW transition planning and implementation (Ministry of Health, Public Service, and Finance) [6]. In addition, we conducted interviews with the representatives of PEPFAR implementing organizations at the coordinating national and sub-national operational levels to understand transition processes that potentially influenced HW retention, such as the initial 2-year orientation phase in GoU service. The complete list of category of respondents is reflected in Table 3. Data were collected over two rounds. For districts which had the highest HW absorption rates (Table 2) interviews were conducted between June and September 2018 (round 1). For districts with the lowest HW absorption rates interviews were conducted between January and March 2020 (round 2) [6].

**Table 3:** Category of participants

Respondent type	Round 1	Round 2	Total
High-level sector ministry technocrats	14	0	14
District Health Team leaders	12	3	15
Facility in-charges/managers	18	4	22
Representatives of regional-based PEPFAR implementing partners (IPs)	11	2	13
U.S. embassy program officers (USAID and CDC)	3	0	3
Focus group discussions	6	2	15
Transitioned health workers	75	12	87

Two authors (HZ and JR) conducted the interviews and were assisted by two research assistants.

### Data analysis

We made audio recordings of all the focus groups and interviews and transcribed both verbatim*.* In terms of our qualitative data analysis procedures, we followed the framework method [21]. More specifically, we followed four significant steps. However, this was a largely iterative process*.* The first step involved data familiarization through multiple readings of transcripts by HZ and JR*.* The second step entailed generating a coding framework. Codes were generated inductively from the transcripts in a team-based process involving two authors (HZ, JR)*.* The third stage was that of abstracting the coded data into thematic categories. The emergent inductive or data-driven codes were then grouped under a deductive thematic framework based on Schaefer and Moos’ [18] lens of individual-level and organizational context influences on employee outcomes. However, we found that community-embeddedness was a recurring theme that was not adequately captured by the adopted analytical framework [18]. Hence, our coding combined both deductive and inductive analysis approaches*.* The fourth and final step was that of overall interpretation and synthesis. This process involved four authors (HZ, JR, MK and WA).

## Results

The findings emerging from this study are presented based on the domains of individual, organizational [18] as well as community-level drivers.

### Individual-level drivers

Individual-level facilitators of retention which emerged from this study are categorized under monetary and non-monetary incentives.

### Monetary facilitators

#### Job security

Overall, job security was identified by HWs as the most important incentive for opting to remain in public sector employment. It was widely perceived that continued employment in the Uganda public service system ensured stable and guaranteed monthly income. Anticipated regular income after retirement through pension earnings was an additional incentive for HWs.

*‘For me I think being absorbed into public service was a blessing because when we were with PEPFAR, we were not sure of job security. But at least now we know we are on a permanent job’* [FGD HWs, South Western Uganda].

Similar sentiments were expressed by a health worker from Eastern Uganda who alluded to how job security facilitated peace of mind and secured long-term employment.

*‘What motivates me number one is job security. When compared to working for an NGO, like I told you, PEPFAR keeps giving you annual rolling contracts. After one year, you keep wondering where I am I going? Am I going back to my rural village? But now, with local government, the job security is there. As long as you perform well, your job employment is guaranteed. It is you to say I am tired’* [FGD HWs, Eastern Uganda].

In our focus groups with HWs, they indicated that the opportunity of having a ‘permanent and pensionable’ job was a cherished one. This was triangulated with data from in-depth interviews from facility managers who concurred that having a public service job is a precious one in Uganda in the context of prolonged delays in recruitment for public sector jobs in Uganda.

Health workers praised the PEPFAR HIV workforce expansion intervention for providing them with an entry point into public service, which many considered rare in Uganda due to prolonged delays in recruitment owing to long-standing wage bill ceilings.

*‘I am happy about the transition because it’s very hard to get a job in the public service in Uganda. So PEPFAR made it easy for us to just ‘cross’* [FGD HWs, Southwestern Uganda].

Another related notion to steady income was the frequently cited advantage of securing salary loans from banks and other financial institutions because of the perceived stability of public sector employment and reliable monthly payroll payments.

*‘You can go to the bank and get a loan…they can deduct it from your salary since it is paid regularly without any interruptions, and that loan can solve other problems, and you can venture into other businesses’* [FGD HWs, Northern Uganda].

*‘Because of the salary loan many of us are able to do some digging and gardening because our land is fertile, and you can borrow or rent some piece of land’* [FGD HWs Central Uganda].

With respect to mid-cadre HWs, particularly nurses and midwives, the public sector often pays more and is seen as a more attractive employment option when compared to the private sector. Indeed, most mid-level health workers cherished the opportunity of finally joining the Ugandan public service and noted they had longed for it.

‘*I feel excited about working for the government of Uganda because this is what I was longing for’* (FGD HWs, Eastern Uganda).

District health teams and national-level ministry officials concurred with HWs in relaying the notion that opportunities for entry in the Ugandan public service were rare and hence public sector jobs were highly prized.

*‘Jobs are not easily available in public service. They are very difficult to get. There are many people out there looking for a job… some of these people they recruited them when they had been on ‘the street’ (unemployed) for a long time looking for the opportunity’* (KII District Official, Eastern Uganda).

*‘Even for us, being employed in public service, it is a motivation factor. Being somehow permanent (in public service). Because when you are on contract, they can say, ‘the contract is not going to be renewed’. So, if there is something worth fighting for, it is being fully recruited and appointed in the public service’* [IDI, District Official, South Western Uganda].

### Community embeddedness

An important driver of health worker retention was the existence of social ties in the districts in which many of the 694 health workers were absorbed. Indeed, most absorbed HWs called these ‘home districts’ or hailed from that broader ethnic sub-region. Health workers expressed satisfaction in being able to work in their home districts, where they could serve their ‘own’ kinsmen. For hard-to-reach areas in parts of select districts (Kasese, Nwoya, Napak and Apac), which experience difficulty in attracting and retaining health workers due to being remote and isolated, health workers from those areas looked at themselves as the only ones willing to serve in those conditions.

*‘People were not willing to come and work here. Different people were employed and did not report for work. So for us, we saw and said that if we do not help our own indigenous people, who will help these people? That is why I am still in the local government otherwise, there were very many different opportunities. South Sudan is just very near, even going to Kampala I would just get a new job’* [FGD, HW Northern Uganda].

Working in home districts or broader sub-regions enabled health workers to supplement their income by tending to food gardens in rural districts and drawing upon their blood relations for financial or in-kind support, such as food provisions, during occasional government salary delays.

*‘Working near home has given me hope of working somehow comfortably because even if you don’t have what to eat, you rush home and get some food supplies’* [FGD, HW South Western Uganda].

Some facility in-charges relayed the notion that ‘local hires’ from the districts were more resilient, with perceived better retention outcomes, compared to HWs recruited from outside the district. The latter was deemed unfamiliar with the ‘local terrain’ in rural districts.

*‘What we have observed over time is that health workers who have rural backgrounds especially those who are natives of this district tend to build resilience in overcoming local hardships compared to those from outside this region of Uganda who give up when the going gets tough because they have no social ties as persistence enhancers’* [IDI, District Official, Northern Uganda].

The perception that HWs who grew up in rural areas would suffer less attrition in rural districts compared to those who didn’t have this background was common among district health team members and facility in-charges.

### Career advancement

Most HWs reported being attracted to remaining in public service due to opportunities for advancing in their careers, given the clear and established structures for promotion and growth in the public health sector. Some of the 86 HWs transitioned from PEPFAR support and absorbed into the public sector payroll had already risen through the ranks in their health facilities of transition through meritorious promotion.

‘*In the public service there is a clear path for rising through the ranks and getting promoted progressively. In private hospitals you can remain on the same rank for ages but when you work in public service you keep growing over the years’* [FGD, HW Northern Uganda].

A medical officer or physician absorbed by a district in Northern Uganda in 2015 had risen to the position of Medical Superintendent or head of the district hospital. In Eastern Uganda, a PEPFAR-recruited laboratory technician had been promoted to the position of head of laboratory services at the district hospital. Another laboratory technician based at a district hospital in Northern Uganda had been assigned to supervise laboratories at lower-level health facilities.

*‘I have been assigned as laboratory sector mentor for this district. I visit the labs of lower health centres to mentor their staff on maintaining good standards and maintenance of their equipment to ensure increased durability’* [FGD, HW Northern Uganda].

Several previously PEPFAR-supported health workers had been designated heads of the ART clinics in health facilities, where they were eventually absorbed.

### Opportunities for further training

Absorbed health workers indicated they opted for service in the public sector owing to what was perceived as the inherent opportunities for in-service training in the form of seminars and workshops. In addition, study participants reported that unlike in the private sector, there are many more opportunities for study scholarships and formal study leaves in the public health sector. Employment in public service was associated with prospects for gaining new skills and additional academic qualifications, which would enhance opportunities for securing new positions in the public service on promotion and, ultimately better pay.

*‘The other issue is that private for-profit facilities have no training. I don’t remember attending any trainings while in private facilities, but ever since I joined the government, there have been so many trainings at the local, regional and national level, and I have interfaced with so many people of various cadres* [FGD HWs, Eastern Uganda].

*‘For me, what has helped me remain with the government is that it has helped me build my career because, like he said, I have attended so many trainings, known friends and met various health workers in the country*. *Uganda’s government is good at capacity building. You came in with a lower level of education but after you are confirmed in service, they can offer you leave and let you go and study, but NGOs it is very rare for them to give you study leave’* [HW FGDs, Northern Uganda].

### Organizational-context drivers

#### Flexibility in public sector work environments

A section of the 86 HWs who participated in our study had private sector work backgrounds. These HWs indicated that in the private health sector, they were under more stringent supervision regimes while noting that the public health sector is a lot less stringent. Key factors for HWs opting to join and remain in government employment included flexibility in reporting time for work, relaxed supervision by superiors, opportunities to reschedule work or exchange work hours with colleagues. Likewise, they noted that public facilities availed spare time to supplement the low salaries paid to HWs by the government. All the preceding observations were critical factors for HWs opting to join and remain in government employment. All health workers disliked strict supervision by the immediate supervisor, timesheets and frequent performance appraisals during the 2-year PEPFAR transition phase. The notion that public service work environments had less-intensive supervision regimes compared to the private sector which was raised in a number of focus groups with HWs was triangulated with reports from district health teams.

*‘They have a lot of freedom in government service. Nobody is watching over their backs so much’* [IDI, District Official, Eastern Uganda].

*‘For me, I like working for the Uganda government because of the liberty you have. In private organizations, you are not allowed to fall sick, you don’t lose someone (bereavement), and you don’t go on maternity leave. When we were under contract, it was tiresome having to sign timesheets, what, what. But now we are free. But we thank them because they are the ones who brought us, and now the government is treating us well*’ [HW FGDs, Western Uganda].

On the other hand, some facility in-charges observed that some of the absorbed PEPFAR workforces had slackened in their work ethic compared to when they were under the contract phase, where performance was very closely monitored.

#### The role of orientation in enhancing HW retention

Absorbed health workers indicated that during their 2-year contract phase under PEPFAR support, they were initiated and oriented into Uganda’s public sector work methods, processes and guidance on personal conduct. The contract phase under PEPFAR enabled health workers to practice at public facilities, where they were to be absorbed. This phase was described as a phase of adjustment to the context characterized by multiple challenging operational contexts at the health facility and the broader environment. At absorption, health workers were familiar with the government work environment, including the constraints common in public facilities, particularly the chronic shortage of essential commodities and heavy workloads to which they had adjusted.

*‘When they came in, orientation was done so that they know the government working system’* [IDI, District Official, Northern Uganda].

We observed variabilities in the long-term service intentions of the cadre of health workers. Mid-cadre staff, such as nurses and midwives, indicated a stronger inclination to continue long-term in the public health sector compared to other health worker cadres. Upper-rung health workers such as physicians and pharmacists demonstrated a higher affinity for seeking alternative employment soon.

*‘You can imagine working for this paltry monthly salary ($285) in this remote and rural part of Uganda where there are no good schools for my children or even a decent bar to go to in the evening. Yet my peers are earning big monies working for foreign NGOs in the capital. Of course when I get a better paying opportunity I will not hesitate to leave’* [HW FGDs, Western Uganda].

For pharmacists and radiographers, district health teams expressed an inability to attract this cadre of health workers despite repeated job adverts and that the few who reported only remained in their posts for a short time.

*‘Our district being in a rural setting, those high calibre cadres like physicians, the pharmacists, it was a challenge to attract and retain those people. For medical officers, we had a very serious gap. It was only recently when we got a pharmacist, and actually for a short time, and he also went away’* [IDI District, Northern Uganda].

#### Quest for challenging professional work

A section of health workers preferred GoU service, because, relative to their work experience in for-profit clinics, working in public facilities offered a more challenging and diverse work experience, enabling them to optimize their competencies and training.

*‘What has made me stay in government is to obtain more experience because I am handling many patients, so many cases compared to private facilities because sometimes we see few patients and become inexperienced. …I have received so many trainings in TB and …it has helped me improve on the knowledge gap, and I have come across so many cases which I didn’t encounter when I was in training*’ [HW FGDs, Eastern Uganda].

#### Work environment barriers to long-term retention

Although participating HWs had been retained in the public health sector for at least 3 years after their official absorption, their long-term intentions to remain in the GoU service were uncertain. At individual level, HWs, particularly for upper cadres such as physicians and pharmacists, identified barriers to long-term retention that include low pay and the availability of better alternative jobs elsewhere.

At the organizational-level, HWs expressed general dissatisfaction with the chronic stock-out of essential commodities common at district hospitals. Basic supplies needed in their routine practice, such as surgical gloves, gauze, and even common essential medicines, were frequently out of stock. HWs indicated this was a constant source of frustration of being unable to optimize their professional competencies to deliver services to patients seeking care.

*‘Inconsistent supplies are a headache. Today supplies are available. Tomorrow they are not there, and yet you can’t work when they are not there. Everything is out of stock, the medicines, the gloves, they can come in and in three weeks and we are out of stock. Supplies which are provided on a quarterly basis only last three weeks’* [FGD, HW, Eastern Uganda].

Furthermore, HWs reported a scarcity of rent accommodation as a barrier to long-term retention in the public sector. The shortage of rent accommodation was especially prominent in rural Northern Uganda. HWs in districts in this region expressed an inability to secure rental housing near district hospitals and having to travel several 100 km away daily to their workstations. This category of HWs indicated a willingness to seek alternative employment when the opportunity presented itself. Some HWs who had hospital-provided staff housing said that overcrowding and congestion were a consistent challenge. HWs described the discomfort of sharing housing units meant for single occupants. In the focus groups, HWs raised poor housing conditions and the critical shortage of rental accommodation in some parts of Uganda as threats to long term retention which was corroborated in our in-depth interviews with district health teams.

*‘Another challenge is accommodation. When you go to housing for HWs at the district hospital, you find four doctors in one house. All HWs at the hospital are sharing. Someone is in the bedroom, and another is sleeping in the sitting room”* [IDI, District Official, Southwestern Uganda].

HWs also voiced concerns about the heavy workloads they were subjected to due to persisting staffing gaps at district hospitals. While several HWs exhibited remarkable fortitude in enduring hardships in district hospitals, a section of HWs expressed a likelihood of leaving if better employment opportunities, with higher pay, became available.

*‘If I get another job, I can leave without even resigning or wasting time simply because the payment is low and irregular. If I get another job with an NGO, I will also leave government employment because with big NGOs pay better’* [HW FGD, Northern Uganda].

Delays in confirmation in public service were frequently cited as a source of demotivation. In several study districts such as Sheema, Iganga and Mubende, HWs expressed unease and insecurity with delays in confirming them in government service. The delay in formal confirmation in public service after completing the 2-year probation period emerged as another threat to long-term retention.

## Discussion

We utilized Schaefer and Moos’ analytical framework of micro and meso-level [18] determinants of employee retention to explore drivers of retention of the health workforce transitioned from PEPFAR support to Uganda government payroll between 2015 and 2017. At the individual level, we found that job security was the most compelling motivation for remaining in the public health sector. Community embeddedness or employee social ties in the districts of deployment, ability to secure salary loans from financial institutions due to perceived stable employment by GoU and opportunities for promotion and career advancement within Ugandan public service ranks were the other drivers of retention of the absorbed workforce.

At the meso or organizational-level, absorbed HWs revealed that relaxed supervision regimes, and being familiar with public health sector organizational cultures and environments following their 2-year orientation in district hospitals with PEPFAR salary support were significant drivers of retention in the public health sector. Unlike previous studies which have examined the retention of in-service public sector health workers [9–12], a section of HWs who participated in this study had private sector work backgrounds. The latter participants’ motivations for retention should be viewed in the context of transition from private to public health sector employment.

While our findings are consistent with previous studies that reported that HW social ties in rural communities enhance retention [22], our study unearths new retention drivers such as HWs’ ability to secure salary loans due to perceptions by lending institutions that GoU employment was stable with guaranteed monthly payments.

A significant finding from our study is the notion by mid-cadre HWs comprising nurses and midwives that there are currently not many competitive alternative opportunities, in terms of pay scales, with employment in public hospitals in Uganda [23]. This may be related to efforts by the GoU over the past 5 years to progressively enhance the salaries of HWs in public facilities to levels that are currently unmatched by most private providers [24]. The pay disparities across public and private facilities perpetuated the notion among absorbed HWs that enrolment onto the public sector payroll was a rare, highly prized opportunity. This was against a backdrop of prolonged delays in recruitment in district health systems owing to ‘wage bill ceilings’ [6, 25]. On the other hand, district health teams and facility in-charges described challenges around the retention of upper-rung cadres, such as physicians, pharmacists and laboratory technologists. As reported in previously published research in Sub-Saharan Africa [26–32], these challenges included chronic shortage of essential supplies and commodities, scarcity of rental accommodation in predominantly rural settings, and heavy workloads due to persistent staffing gaps. The GoU ought to deploy monetary and non-monetary interventions to enhance retention outcomes for these cadres, such as providing additional financial incentives for working in rural or hard-to-reach areas as is provided in select parts of Uganda at the level of sub-district hospitals in Uganda. Our recommendations align with previous studies that identified incentives to improve the work environment in resource-constrained settings [33–35]. Our findings further emphasize the necessity for expanding hospital-provided HW accommodation in rural Uganda, where a shortage of rent accommodation inhibited work efficiency by health workers.

In reflecting on the analytical framework by Schaefer and Moos [18], we found utility in this approach in eliciting individual-level motivation for remaining in the public health sector, such as the desire for financial stability as well as organizational context factors such as HWs being familiar with work cultures in public facilities in Uganda. However, we found dynamic interactions between the two clusters of drivers at the micro and meso levels. In addition, we found that the analytical framework we selected [18] did not adequately capture the recurring theme of HW community-embeddedness. More specifically, we found that HWs leveraged social ties for food and tended to gardens in their ‘home districts’ during delays in monthly salary payments or long, drawn-out processes for enrollment on the public sector payroll. Furthermore, adding a complex adaptive health system [36] lens in understanding HW retention could be helpful in exploring intra-personal adaptive capacity in navigating challenging operational contexts in rural Uganda.

With respect to the transition processes of HWs initially recruited under GHI frameworks, we found that our Ugandan case study suggests that (1) aligning with donor–recipient government systems such as salary scales for HWs, working within GoU recruitments mechanisms particularly at the sub-national level and (2) providing a 2-year orientation phase enhanced both the HIV workforce transition process itself but also the adaptation of the absorbed HWs to public sector work environments. Our study corroborates previous studies [37–42] calling for aligning donor transitions and aid mechanisms with beneficiary country policies and structures in the health sector.

An important finding of this study was the emerging notion that the community embeddedness of HWs, especially those with rural backgrounds, enhances HW retention in rural districts of Uganda. Participants reported that HWs with rural backgrounds are more likely to leverage their social networks such as securing food during salary delays by leaning on relatives or tending their food gardens in their backyards and hence, are more likely ‘to stick it out’ at public facilities. Indeed, there is gathering momentum around the case for preferential treatment of candidates with rural backgrounds as a pragmatic strategy for enhancing HW retention in rural areas [14, 43]. Russell et al. [14] call for pre-service tertiary institutions to integrate extended rural training as a part of HW training to improve retention outcomes in rural and remote settings [44, 45].

Our study had multiple limitations. It was a challenge to untangle the drivers of retention due to the dynamic interactions in these factors and intersecting drivers at various levels (such as at the individual and community levels) which influence retention of health workers in the Ugandan public health sector making it difficult to precisely attribute outcomes to any single driver. Another potential limitation was desirability bias. Previously PEPFAR-recruited health workers may have wished to provide responses that they feel would put them in good stead and to project gratitude for the opportunity extended to them to join Ugandan public service ranks.

## Conclusion

Overall, job security was the most compelling driver of retention in public service for the HIV workforce transitioned from PEPFAR support to the Uganda government payroll. However, HW retention in case-study districts was embedded in a complex system involving individual-level (eligibility for salary loans, quest for fulfilling professional work), community-level (social ties in intervention districts, opportunity to serve in ‘home’ districts) and organizational context drivers (less stringent supervision regimes, relaxed work environments).

Monetary and non-monetary policy strategies are needed to enhance the retention of upper cadre HWs, particularly physicians, pharmacists and laboratory technologists in rural districts of Uganda.

## Data Availability

The data sets generated during and/or analyzed during the current study are not publicly available due to ethical reasons but are available from the corresponding author on reasonable request.

## Abbreviations

AIDS: Acquired immune deficiency syndrome
ADRs: Adverse drug reactions
ART: Anti-retroviral therapy
HW: Health worker
MOH: Ministry of Health
PEPFAR: The Presidents’ Emergency Plan for AIDS Relief
PLHIV: People living with HIV
SSA: Sub-Saharan Africa
WHO: World Health Organization
